# No difference in the long term final functional outcome after nailing or cast bracing of high energy displaced tibial shaft fractures

**DOI:** 10.1186/1752-2897-6-5

**Published:** 2012-06-13

**Authors:** Vineet Batta, Amitabh J Dwyer, Aashish Gulati, Jeevan Prakash, Maharaj K Mam, Bobby John

**Affiliations:** 1 Department of Orthopedics, Christian Medical College & Hospital, Brown Road, Ludhiana, 141008, Punjab, India

**Keywords:** Fracture shaft tibia, Treatment methods, Plaster cast brace, Intramedullary nail

## Abstract

**Background:**

Cast bracing (CB) has been a well established method of treating tibial shaft fractures. Majority of the recent literature on treatment of tibial shaft fractures have upheld intramedullary nailing (IMN) as the treatment of choice. Most of these studies are from the west, in public funded health set ups and in hospitals with very low rates of infection. This has lead to bewilderment in the minds of surgeons wishing to opt for conservative treatment in countries with scarcity of health resources. We therefore undertook this study to compare the two modalities in the scenario of the developing world.

**Material and methods:**

Sixty-eight consecutive patients were treated alternately with CB and IMN for high energy, displaced, closed and Gustilo Grade 1 open fractures of the tibial shaft, between 1995 and 2001.

**Results:**

An average follow up at 4.3 years revealed no statistical difference in the final functional outcome as per Johner and Wruhs' criteria with modification to Indian lifestyle. IMN group had a) slightly shorter time to fracture union (mean 21.3 weeks versus 23.1 weeks for CB, p > 0.05), (b) lesser time off work (mean 17.6 weeks versus 25.6 weeks for CB, p <0.01), (c) fewer outpatient visits (mean 6.2 versus 9.7 for CB, p < 0.05), (d) less limb length discrepancy (mean 4.3 mm versus 6.6 mm for CB, p < 0.05). The difference in residual antero-posterior angulation (mean 3.2 degrees for IMN versus 4.9 degrees for CB, p = 0.14) and varus-valgus angulation (mean 3.7 degrees for IMN versus 5.1 degrees for CB, p = 0.7) were not statistically significant. However CB group had no deep infections as compared to two in the IMN group. The average cost of hospital treatment of CB group was less than half incurred by the IMN group (average USD 831 versus USD 2071 for nailed group, p < 0.05).

**Conclusion:**

Treating tibial shaft fracture either with IMN or CB provided equally gratifying results with no statistical difference in final functional outcome. The economic cost to the patient in Indian conditions is significantly less with CB and therefore stands as an equally reliable treatment option, especially in countries with fewer resources.

## Background

Tibia is the most commonly fractured long bone in the body [1]. Cast bracing (CB) of diaphyseal tibial fracture has shown success since its use from 1960’s [2,3]. It harvests the potential of the soft tissue for fracture stabilization by constraining them within a shell of brace, thus creating a hydraulic pressure-like effect that stabilizes the fracture [3,4]. The controlled motion allowed by the brace at the fracture site is conducive to osteogenesis [5]. However, a displaced unstable tibial shaft fracture poses difficult problems to even an experienced surgeon [6]. Intramedullary nail (IMN) bears the advantage that it reduces and stabilizes the fracture simultaneously [7] , albeit with the penalty of increased surgical risks [8], chronic insertion site pain, nail protrusion and a potential need for a second procedure for hard ware removal [9,10].

In spite of the frequent occurrence and clearly established goals of treatment, i.e. restoration of anatomy and early return to function, the best treatment still remains controversial [5,8,9,11]. Large differences exist across the health systems of the West and the developing world. Indian health system is largely driven by fee-based service of the private sector. Hence, cost is one of the major considerations both to the patient and the surgeon when deciding the choice of treatment.

We analyzed the functional outcome and cost effectiveness of cast bracing(CB) versus intramedullary interlock (IMN) nailing of closed, displaced and Gustilo Anderson Type 1 open fractures of tibial shaft [12] in adults.

## Material & methods

This prospective study was conducted in the Department of Orthopedics, Christian Medical College, a tertiary medical centre located in Ludhiana, Punjab, India. The study period extended from between 1^st^ May 1995 and 30^th^ April 2001. Prior approval was sought from the Institutional Ethics Committee and all investigations were conducted in conformity with the declaration of Helsinki. An informed written consent was obtained from all the participants included in the study.

Adult patients presenting with unilateral tibial fractures within 6 hours of injury were included in the study. The inclusion criteria were strictly limited to unilateral closed and Grade 1 open tibial shaft fractures in a zone extending from 5 cm below the knee up to 5 cm above the ankle with a fracture displacement of ≥50 % and an angulation of >10° in antero-posterior or lateral views [13]. Patients with grade 2 or 3 open fractures and those with concomitant long bone injuries were excluded.

### Treatment

Grade 1 open fracture wound was debrided, irrigated and closed primarily within 6 hours of presentation. The patients then received prophylactic broad-spectrum antibiotics as per the hospital policy. Patients were alternatively treated by either of the two modalities, *viz* cast bracing (CB) or intramedullary nailing (IMN). Sushrut (Made in India) or Zimmer cannulated nailing system was used depending upon the affordability of the patient. Wounds were inspected after 48 hours on the ward and weight bearing using elbow crutches commenced as dictated by the fracture pattern, size of the nail and associated injuries. In the IMN group the tibial fracture was managed using an ‘*unreamed intramedullary nail*’. All cases were routinely immobilised in an above knee plaster of Paris slab till active dorsiflexion of the foot was possible, usually by the second/third day

Patients in the CB group were treated with an ‘*above-knee touch weight bearing plaster*’ for six weeks followed by ‘*below-knee cast brace*’ while they were encouraged gradual progressive weight bearing.

### Follow up

The patients were assessed by an independent researcher who did not participate in the treatment of these patients. Cases were followed up clinically and radiologically at 6 and 12 weeks post surgery, and then every 6 weeks till consolidation of the fracture and thereafter, every three months provided the patient were asymptomatic. The fracture was considered united when the patient was able to walk unaided, bearing full weight through the affected lower limb without pain in addition to the radiographic evidence of union [14]. The final functional outcome was assessed using the modified ‘*Johner and Wruhs’ criteria with modification*’ to suit life style needs for an Indian patient (Table 1). Fracture that did not unite radiographically by 26 weeks was considered ‘*delayed union*’ and a ‘*non union*’ was accepted as absence of radiographic union by 9 months [14]. The fracture was considered to have ‘*malunited*’ if there was >5° of varus or valgus and an anterior/posterior angulation >10° [14].

**Table 1:** Johner and Wruh’s (1983) criteria with modification

**Criteria**	**Excellent**	**Good**	**Fair**	**Poor**
**Nonunion/infection**	None	None	None	Yes
**Neurovascular injury**	None	Minimum	Moderate	Severe
**Deformity**				
**Varus/valgus**	None	2-5^0^	6-10^0^	>10^0^
**Ante/Posterior**	0-5^0^	6-10^0^	11-20^0^	>20^0^
**Shortening**	0-5 mm	6-10 mm	11-20 mm	>20 mm
**Mobility**				
**Knee**	Full	>90 %	90 - 75 %	<75 %
**Ankle**	Full	>75 %	75-50 %	<50 %
**Pain**	None	Occasional	Moderate	Severe
**Gait**	Normal	Normal	Mild limp	Significant limp

### Cost analysis

A detailed analysis of the cost of treatment in the two groups was undertaken. The costs caused by the direct costs (treatment, hospitalisation, and outpatient appointments) as well as indirect costs (lost productivity) were taken into account. Costs caused by complications were also included in the analysis.

### Statistical analysis

The data was analyzed using SPSS version 13.0 (Chicago, IL, USA). Demographic characteristics of participants in both groups were compared to ascertain any confounding factors. Chi-Square or Fischer’s exact test was used for categorical variables and student’s t test (2-tailed) for quantitative variables. P-value of less than 0.05 was taken to be statistically significant.

## Results

Between 1^st^ May 1995 and 30^th^ April 2001, a total of 419 patients with fracture of tibial shaft were treated in the Department of Orthopedics, Christian Medical College, Ludhiana, India. Of these, 287 patients had open fractures (Gustilo Grade 1: 62, Gustilo Grade 2: 106, Gustilo Grade 3: 119) and the remaining 132 patients had a closed fracture (displaced: 85, undisplaced: 47). There were 68 patients who satisfied our inclusion criteria and were thus recruited in our study (closed fracture: 49, Grade 1 open fracture: 19). Majority of the patients were males (n = 60, 88 %). Both the groups were comparable for age, gender, mode of injury and the type of fracture (Table 2). Majority of patients were aged between 15 and 34 years of age (n = 33, 48.5 %). The most common etiology of fracture was road traffic crashes (n = 59). Most of the patients injured were riding a two-wheeler at the time of sustaining the injury (n = 30), this was followed by fall from heights and scaffoldings (n = 6), and physical assault (n = 3). Right extremity was more commonly injured (62 %) and was observed frequently among motorcyclists colliding with the oncoming traffic from the right side. Oblique and spiral fractures were common (82.4 %). Fractures most commonly occurred in the middle and the distal third of the shaft of tibia (97.1 %). Consecutive patients were alternatively treated with the two modalities; 34 patients were managed by cast bracing (CB group) and 34 patients were managed by intramedullary nailing ( IMN group).

**Table 2:** Demographic characteristics of patients in both groups

	**Cast bracing group(n = 34)**	**Intramedullary nailing group (n = 34)**
**Age (yrs) (Mean ± SD)**	36.2 ± 15.4	39.3 ± 13.9
**Age group** < 25 years 25-50 years ≥50 years	5 20 9	422 8
**Sex (n)** Male Female	31 3	28 6
**Mode of Injury (n)** Assault Fall Road traffic accident	2 3 29	1 3 30
**Type of tibial fracture (n)** Transverse Spiral Oblique	7 11 16	5 12 17
**Level of fracture in shaft of tibia** Proximal third Middle third Distal third	1 14 19	1 18 15

### Comparision of Outcome Measures (Tables 3 and 4)

**Table 3:** Outcome of patients in both groups

	**Cast bracing group (n = 34)**	**Intra-medullary nailing group (n = 34)**	**P value**
**Clinical Outcome**			
**Duration of hospital stay** (days) *Mean ± SD*	4.2 ± 4.8 days	6.1 ±5.1 days	0.1185*
**Time to union***Mean ± SD*	23.1 ± 8.5	21.3 ± 11.1	0.4557*
**Time to return to work** (weeks) *Mean ± SD*	25.6 ± 7.3	17.6 ± 9.8	0.0003*
**Out-patient visits***Mean ± SD*	9.7 ± 1.7	6.2 ± 1.3	0.0001*
**Radiological Outcome**			
**Varus/valgus angulation** (degrees) *Mean ± SD (Range)*	5.1 ± 3.4 (1-15)	3.7 ± 2.9 (0-15)	0.0723*
**Antero-posterior angulation** (degrees) *Mean ± SD (Range)*	4.9 ± 5.5 (2-30)	3.2 ± 3.8 (0-20)	0.1429*
**Limb length discrepancy** (mm) *Mean ± SD (Range)*	6.6 ± 4.6 (0-18)	4.3 ± 4.7 (0-8)	0.0454*
**Final Functional Outcome**			
**Functional outcome**Excellent *(n, %)* Good *(n, %)* Fair *(n, %)* Poor *(n, %)*	3 20 8 3	5 19 6 4	0.829**
**Gait following treatment** Normal Mild limp Significant limp	21 7 4	28 4 2	0.374**
**Able to squat**	30	31	1.00**
**Able to run**	19	21	0.662**
**Additional procedure needed** Bone grafting Re-reduction Fibulectomy Nail removal	3 5 1 0	4 0 2 5	1.000** 0.053** 1.000** 0.053**

*Unpaired t-test ** Fisher’s Exact test

**Table 4:** Complications during treatment in both groups

**Complication**	**Cast bracing group (n = 34) ( %)**	**Intramedullary nailing group (n = 34)( %)**	**P Value (Fisher’s exact test)**
**Delayed union**	8(23.52 %)	2(5.8 %)	0.083
**Malunion**	10 (29.4 %)	5 (14.7 %)	0.242
**Non-union**	1 (2.9 %)	3 (8.8 %)	0.614
**Deep Infection**	0 (0.0 %)	3 (8.8 %)	0.239
**Anterior Knee pain**	1(2.9 %)	20(58.8 %)	0.000 (p < 0.001)
**Persistent leg edema**	3(8.8 %)	1(2.9 %)	0.614
**Deep vein thrombosis**	1(2.9 %)	2(5.8 %)	1.000
**Pulmonary embolus**	0(0.0 %)	1(2.9 %)	1.000
**Clawed toes due to occult compartment syndrome**	0(0.0 %)	1(2.9 %)	1.000
**Limb length discrepancy**	5(14.71 %)	2(5.8 %)	0.427
**Peroneal nerve palsy**	0(0.0 %)	2(5.8 %)	0.493

The mean duration of follow up was 36.6 ± 20.3 months in the CB group and 44.4 ± 18 months in the IMN group. The difference in the duration of hospital stay, time to union for the two groups was not statistically significant (Table 3). The patients in IMN group returned to their profession at a mean of 8.03 weeks (p < 0.05) earlier as compared to CB group (Table 3). The CB group had an average 3.5 out-patient visits (p < 0.05) more that the IMN group. Radiological observations of angulation deformities in sagittal and coronal planes at final follow up showed slightly greater deformities in the CB group (Table 3) but the difference was not significant. On clinical examination, a mean shortening of 4.3 mm was observed in IMN group as compared to 6.6 mm in CB group (p < 0.05). About 40 % of fractures of IMN group had shortening of less than 2 mm as compared to 18 % of CB group. Four fractures in IMN group (Table 4) needed bone grafting for non-union (n = 3) and delayed union (n = 1) and they united at 42, 56, 64 and 32 weeks respectively. In contrast, 3 fractures in CB group required bone grafted for a delayed union which united at 39, 44, and 53 weeks respectively. The final functional outcome measured by Johner and Wruhs’ [13] criteria with modification showed 23 (67.6 %) and 24 (70.6 %) excellent to good results in CB and IMN patients respectively (Table 3). Though, the functional outcome of IMN group appeared more favorable, it was not statistically significant (*p > 0.05* ).

### Complications (Table 4)

Complications were observed in both groups, though they were more in the IMN group compared to the CB group. Hardware failure occurred in seven nails that included a broken nail, a bent nail, two broken screws and loosening of three screws. Twenty patients (59 %) with intra-medullary nailing had varying degree of anterior knee pain; of these 9 (26.5 %) had pain on kneeling while seven (20.6 %) had pain on strenuous activities. Four patients (11.7 %) had severe anterior knee pain and nail removal helped in complete and partial relief of pain in two patients respectively. One patient in the IMN group developed deep infection requiring removal of nail after fracture union. One CB group patient had clawing of toes probably due to occult compartment syndrome of deep posterior compartment of the leg.

### Cost analysis

Mean direct cost per patient in the CB group was INR {Indian rupees} 33,240/ USD 831{One USD = 40 INR}) and in the IMN group was INR 82,931/ USD 2073.2 (p < 0.05). Total economic loss was calculated taking into account the average per capita income of the state of Punjab for the year 2003 [15] and the total time off work for each of the groups. The average total cost of treatment, including the economic loss to the patient for the time lost out of work, was 1852 USD and 2383 USD for CB and IMN groups respectively (*p < 0.05*).

## Discussion

Fractures of the tibial shaft occur commonly in the young through their most economically productive years of life. Therefore, prompt and appropriate treatment is essential to curtail prolonged morbidity and disability. Road traffic crashes are the commonest mode of injury. The proportion (80.9 %) in our study is far greater than reported earlier [16]. This could be attributed to the increase in traffic on limited infrastructure of the cities in the developing world, especially in the last decade and a half. Falls from heights and scaffoldings comprise the next common mode of injury and reflect the upsurge in the construction site crashes in the developing world, much in contrast with sports injuries in the western world [14]. Most fractures occurred, either in the middle or distal third of the shaft of tibia and this is in consonance with previous observations [9].

The shorter duration of hospital stay of CB group patients (Table 3) was probably due to early reduction of fracture in the above knee plaster that was applied as soon as the swelling in the leg subsided. Whereas, IMN patients were delayed for operation or discharge due to fear of post-operative infection from the abrasions or wounds in the involved extremity (9/34 patients). This observation is comparable to earlier studies (8,9). Our hospital stay duration are more than those reported in other western studies( 17) as the physiotherapy services were not readily available in the rural areas and so our patients preferred to stay as in-patient till they were comfortably mobilizing with crutches.

Fracture united sooner (Table 3) in the IMN group possibly due to early weight bearing while only touch weight bearing was permitted among CB patients for initial 6 weeks in an attempt to prevent loss of fracture reduction. These observations are similar to fracture union times of 18 [4] and 23.3 [17] weeks reported for patients treated with intramedullary nails.

The mean time off work (Table 3) was shorter for IMN group and they returned to work before complete union of the fracture as they could fully weight bear with the nail in situ. In contrast CB group patients took an average of 1.5 weeks after fracture union to return to work probably due to ankle stiffness and muscle wasting of the limb while in plaster.

Out-patient visits were more for CB group patients as compared to IMN group (Table 3) due to weekly radiographs to confirm maintenance of reduction in initial stages and more physiotherapy visits later to overcome any stiffness of joints following cast immobilization.

Anterior knee pain was the commonest complication (20/34) in IMN group and this offset the otherwise excellent final functional outcome in IMN group. Keating et al. [18] also reported a high incidence (58 %) of anterior knee pain and proceeded to extract the intramedullary nails after 32 months of insertion. They reported complete relief of pain in 45 % of patients, partial relief in 35 % while 20 % had no improvement. Two of our patients each had complete and partial relief of pain after nail removal. It has been suggested that a para-patellar approach and prevention of injury to [1] patella while insertion of IMN may prevent this complication [5,19].

Our analysis of the cost of treatment for the CB group was significantly less than for IMN group. The total cost of treatment including the economic loss to the patient, for the time lost out of work, was also less for the CB group as compared to IMN group (CB:INR 74,080/ USD 1852 and IMN:INR 95,320 / USD 2383). This demonstrates a lesser economic burden on the patient when treated with cast bracing. As 58/68 of our patients were self-employed farmers and their farms were being run by other family members as a result these patients did not suffer significant economic loss during the extra few weeks spent in a cast brace. However, we did not include the cost of complications in the IMN group eg infection, removal of hardware etc. Our results are in contrast with studies from the west. Downing et al. [20] published a detailed analysis comparing the costs of treating tibial diaphyseal fractures treated in a cast or locked intramedullary nailing in UK. The cost was evaluated in terms of the in-hospital costs and the overall costs, taking into account time off work as well. They found that the mean hospital costs were GBP 2226 for plaster treatment and GBP 3727 for intramedullary nailing (significantly different, p < 0.05). The mean time off work was 9 weeks longer in the plaster group and when the cost of lost production through time off work was added to the hospital costs, the overall costs of plaster treatment and intramedullary nailing were GBP 6810 and GBP 6592 (difference not significant). This study suggested that the cost to the hospital of treating these fractures is less with plaster treatment but that the overall cost to the community is no different.

Toivanen et al. [16] compared the relative costs of treating tibial shaft fractures either with a plaster cast or with intramedullary locking nail. They analysed 26 fractures treated in a plaster cast and 51 fractures treated with an intramedullary locking nail. The direct costs (treatment, hospitalisation, and outpatient appointments) as well as indirect costs (lost productivity) were taken into account. Costs caused by complications were also included in the analysis. They concluded that the mean direct costs per patient were USD 4354.8 and USD 5120.8 and mean overall costs per patient were USD 22892.3 and USD 15622.6 in plaster cast and IMN groups, respectively. The higher mean overall costs of the plaster cast group were attributable to the longer sick leave periods in this group (218 days in plaster cast group and 124 in IMN groups). They concluded that plaster cast treatment of simple and spiral wedge tibial shaft fractures requiring closed reduction under anaesthesia is more expensive to society than operative treatment with intramedullary locking nail.

In our opinion, our estimates are in contrast to the western observations as most services (surgeon’s fee and anesthetist’s fee and charges for hospital bed, radiographs, medication, outpatient visit cost) are a lot cheaper in the developing world.

The strength of this study is in the long period of follow up and that no patient was lost to follow up. Both the groups were closely matched. However, we do recognize the fact that was not a randomized study. Though this study has a significant power, the numbers of patients are small to draw sweeping conclusions. We suggest that hospitals which get a large number of trauma should conduct a prospective, randomized and multicentric study.

In conclusion, follow up analysis revealed no statistical difference in the final functional outcome between the two modalities of treatment. Superior ability of intramedullary nailing to achieve a good reduction and to retain the fracture in functionally favorable conditions until healed, simpler follow up, fewer outpatient visits and no need for repeated plaster changes favored it as a preferred method of treatment. However treatment with cast bracing lays less economic burden on the patient, is free of surgical complications and gives equally gratifying results and therefore a good modality of treatment especially in developing countries. Patients should be provided this evidence at time of admission to help them make an informed decision about their preferred treatment.

## Competing interest

The authors declare that they have no competing interests.

## Authors’ contribution

VB: Lead author of the study and operating surgeon. AJD: Statistical analysis and data collection. AG: statistical analysis and data collection. JP: manuscript prepration. MKM: Operating surgeon and Senior supervisor of the study. BJ: Senior supervisor of the study. All authors read and approved the final manuscript.
